# Subgroup Economic Analysis for Glioblastoma in a Health Resource-Limited Setting

**DOI:** 10.1371/journal.pone.0034588

**Published:** 2012-04-12

**Authors:** Bin Wu, Yifeng Miao, Yongrui Bai, Min Ye, Yuejuan Xu, Huafeng Chen, Jinfang Shen, Yongming Qiu

**Affiliations:** 1 Clinical Outcomes and Economics Group, Department of Pharmacy, School of Medicine, Shanghai Jiaotong University, Renji Hospital, Shanghai, China; 2 Neuroscience Center, Nanjing Medical University, Wuxi Second People's Hospital, Shanghai, China; 3 Department of Radiotherapy, School of Medicine, Shanghai Jiaotong University, Renji Hospital, Shanghai, China; 4 Department of Oncology, School of Medicine, Shanghai Jiaotong University, Renji Hospital, Shanghai, China; 5 Department of Oncology, Medical School of South East University, The Second Hospital of Nanjing, Nanjing, China; 6 Department of Neurosurgery, School of Medicine, Shanghai Jiaotong University, Renji Hospital, Shanghai, China; Wake Forest University, United States of America

## Abstract

**Background:**

The aim of this research was to evaluate the economic outcomes of radiotherapy (RT), temozolomide (TMZ) and nitrosourea (NT) strategies for glioblastoma patients with different prognostic factors.

**Methodology/Principal Findings:**

A Markov model was developed to track monthly patient transitions. Transition probabilities and utilities were derived primarily from published reports. Costs were estimated from the perspective of the Chinese healthcare system. The survival data with different prognostic factors were simulated using Weibull survival models. Costs over a 5-year period and quality-adjusted life years (QALYs) were estimated. Probabilistic sensitivity and one-way analyses were performed. The baseline analysis in the overall cohort showed that the TMZ strategy increased the cost and QALY relative to the RT strategy by $25,328.4 and 0.29, respectively; and the TMZ strategy increased the cost and QALY relative to the NT strategy by $23,906.5 and 0.25, respectively. Therefore, the incremental cost effectiveness ratio (ICER) per additional QALY of the TMZ strategy, relative to the RT strategy and the NT strategy, amounts to $87,940.6 and $94,968.3, respectively. Subgroups with more favorable prognostic factors achieved more health benefits with improved ICERs. Probabilistic sensitivity analyses confirmed that the TMZ strategy was not cost-effective. In general, the results were most sensitive to the cost of TMZ, which indicates that better outcomes could be achieved by decreasing the cost of TMZ.

**Conclusions/Significance:**

In health resource-limited settings, TMZ is not a cost-effective option for glioblastoma patients. Selecting patients with more favorable prognostic factors increases the likelihood of cost-effectiveness.

## Introduction

Glioblastoma (GBM) is the most common and most aggressive malignant brain tumor, and it is associated with poor prognoses [1]; The median survival for newly diagnosed GBM cases is less than one year. Most patients will die within two years, and only 12% of patients survive for five years [2]. For newly diagnosed GBM, the current standard of care includes surgical resection to the extent feasible, followed by radiotherapy and adjuvant chemotherapy. Nitrosourea agents are widely administered. Numerous clinical trials have been conducted to investigate the efficacy of adding various chemotherapeutic regimens to radiotherapy [3]. No significant survival benefit was achieved in randomized phase 3 trials testing the combined strategy of nitrosourea-based adjuvant chemotherapy and radiotherapy compared to a strategy involving radiotherapy alone, but in some studies, more long-term survivors were observed in the combined strategy. One meta-analysis based on 12 randomized trials indicated a relatively small survival benefit for the combined strategy over radiotherapy alone (i.e., the survival rate at two years increased by 5%, from 15% to 20%) [4]. These results suggest that adjuvant chemotherapy may have a role in the treatment of newly diagnosed GBM. The unsatisfactory prognosis of GBM indicates a clear medical need for new treatments.

Temozolomide (TMZ) is a new orally administered systemic alkylating agent that crosses the blood-brain barrier (BBB) to exert antitumor activity [5], [6]. The European Organisation for Research and Treatment of Cancer (EORTC) and the National Cancer Institute of Canada (NCIC) Clinical Trials have demonstrated that adjuvant TMZ with radiotherapy significantly prolonged the median progression-free survival (hazard ratio: 0.56, 95% CI: 0.47–0.66; p<0.0001) and the overall survival (hazard ratio: 0.63, 95% CI: 0.53–0.75; p<0.0001) throughout 5 years of follow-up compared to radiotherapy alone. Patients in some favorable prognostic subgroups, such as those aged <50 years and those who are O^6^-methylguanine-DNA methyltransferase (MGMT) methylated, gained more survival benefits [7]. TMZ is currently recommended as a first-line adjuvant chemotherapy in many countries. However, the substantial cost of TMZ restricts its widespread use, especially in health resource-limited regions like China. Nitrosourea agents, such as carmustine, lomustine and nimustine, are still commonly prescribed for patients with glioma because of their relatively lower costs compared to TMZ. Economic studies analyzing the cost-effectiveness of TMZ plus radiotherapy for the treatment of GBM, compared to strategies involving nitrosourea agents and radiotherapy alone, are needed in health resource-constrained settings. Given the obvious differences in survival rates among prognostic subgroups, such cost-effectiveness analysis should be performed to guide clinical practice.

In this economic study, we investigated the 5-year economic outcomes of three first-line strategies for newly diagnosed patients with GBM in both overall and subgroup cohorts based on Chinese clinical practice and recommendations: radiotherapy alone, nitrosourea agents plus radiotherapy and TMZ plus radiotherapy. The follow-up times of most clinical trials have not focused on the 5-year course of the disease despite the previously mentioned paucity of head-to-head comparisons of different strategies. Thus, mathematical modeling techniques must be used to provide information for decision-making. The perspective of the Chinese healthcare system was adopted to assist in determining the direct economic value of the three different first-line strategies in newly diagnosed GMB and to compare the strategies with different willingness-to-pay thresholds per quality-adjusted life-year (QALY) gains. The analysis excluded indirect societal costs (e.g., productivity or caregiver costs).

## Methods

### Analytical Overview

Using the R software package (version 2.13.1; R Development Core Team, Vienna, Austria), a state-transition (Markov) model for newly diagnosed GBM was developed to track the 5-year disease course. We used this model to measure and compare the 5-year direct medical costs and health outcomes for the different first-line strategies for newly diagnosed GBM. The analysis was conducted from the perspective of the Chinese healthcare system. In the model, future costs and health outcomes were not discounted because most survival outcomes among GBM patients are shorter than 2 years.

Patients with different prognostic factors have significantly different survival times. Because no detailed clinical information was available, simulation methods were used to generate the survival rates of patients with combinations of different risk factors. In the current analysis, the prognostic factors that were included were age (<50, 50–60 or >60 years), methylation status of the MGMT promoter (methylated or unmethylated), surgery type (complete resection, partial resection or biopsy) and treatment strategy (radiotherapy alone, radiotherapy plus nitrosourea or radiotherapy plus TMZ). The Markov model developed for the study was used to analyze the cost-effective outcome for each risk subgroup cohort.

Although new therapies for GBM have been evaluated in clinical trials, the most commonly used first-line strategies (after neurosurgery) are radiotherapy alone, radiotherapy plus nitrosourea, radiotherapy plus Gliadel Wafer and radiotherapy plus TMZ [8], [9]. Because the Gliadel Wafer is not supplied in the Chinese market, this analysis evaluated and compared the costs and effectiveness of the following three first-line strategies: radiotherapy alone, radiotherapy plus nitrosourea, and radiotherapy plus TMZ. Because no head-to-head clinical trials have compared these three first-line strategies, an indirect comparison was performed following a well-established approach [10].

Parameter inputs for the model were: transition probabilities (which reflect the probabilities at each cycle of changing between two health states); event proportions (which govern the ratios of events); direct medical costs (which were estimated based on direct health resource consumption); and health state utilities (which project the health-related quality of life for discrete health states). These data were derived from published studies or from local health systems.

Cost-effectiveness ratios were measured to evaluate the outcomes of the different strategies. The main health outcomes were presented with respect to quality-adjusted life-years (QALY). Cost were converted into US dollar (2011 exchange rate, $ 1 = CYN 6.50).The results are presented as an incremental cost-effectiveness ratio (ICER).

### Decision Model Structure

**Figure 1:**
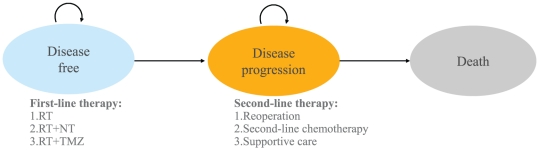
Markov diagram of health states and the possible transitions among them during each 1-month cycle.

A Markov decision model was used to evaluate the 5-year clinical and economic outcomes associated with GBM and its treatment. The cost-effectiveness model for glioma consists of three mutually exclusive health states: disease-free, disease progression and death (Figure 1). In the Markov model, the cycle length was one month, and the patients began in a disease-free state. During each 1-month cycle, patients either remained in the same health state (a recursive arrow) or progressed to a new health state (a straight arrow).

A hypothetical cohort that was clinically similar to the GBM patients in the EORTC-NCIC trial was entered in the model. The hypothetical patients were aged 18–70 years and had newly diagnosed and histologically proven GBM. The patients had a WHO performance status of 0–2 and adequate hematologic, renal, and hepatic functions. Following biopsy or surgical resection, they would receive one of three competing treatment strategies to manage their newly diagnosed GBM (first-line therapy): 1) radiotherapy (RT strategy); 2) radiotherapy and TMZ (TMZ strategy); or 3) radiotherapy and nitrosoureas (NT strategy). When the disease progressed, the patients were treated with a second-line therapy (i.e., surgery, chemotherapy, or a combination of the two) or with the best supportive care (BSC). The second-line chemotherapy strategy was assumed to involve the PCV (procarbazine, lomustine, and vincristine) regime [11].

### Clinical Data

Transition parameters and proportions were derived from randomized clinical trials or meta-analyses whenever possible. Two-parameter Weibull survival models were fitted to the data extracted from the Kaplan–Meier survival PFS and OS curves using R statistical software. Estimated scale and shape parameters and their standard errors (SEs) are listed in Table S1.

PFS and OS survival data for RT and RT+TMZ were derived from the clinical trials [7], [12], [13], whereas survival data for RT+NT were derived from a meta-analysis reported by Stewart et al. [4]. In the two studies, RT was a common control strategy for newly diagnosed GBM, in contrast to RT+TMZ or RT+NT. We assumed that the survival rates from these studies could be compared because the baseline characteristics for the study cohorts were almost identical. To minimize bias, we assumed that the survival rates of RT in the EORTC-NCIC trial were also the baseline in the comparison with RT+NT. We assumed that the survival models for RT and RT+NT could be fitted by Weibull proportional hazards models. As such, the shape parameters of RT+NT were equal to those of RT from the EORTC-NCIC trial. The hazard ratio (HR) between RT and RT+NT that was used to estimate the scale parameters was calculated using the following equation: HR×γ_RT (EORTC-NCIC)_. The HR values between the RT and RT+NT treatment arms were derived from studies reported by Stewart et al. [4]. We assumed that severe adverse events (SAEs) did not change the risk of tumor progression. The proportions of second-line therapy for progressed GMB were derived from EORTC-NCIC [7]. We assumed that the second-line treatment in the RT+NT strategy was similar to that in RT+TMZ.

PFS data were absent for some subgroup cohorts. We assumed that the ratios of the hazard rate between OS and PFS in the overall cohort were equal to those of the subgroup cohorts at any time. Therefore, the absent Weibull parameters for PFS in some subgroup cohorts could be estimated by the following three steps: first, with the Weibull hazard function, we calculated the ratios of the hazard rate between OS and PFS at each cycle in the overall cohort; second, the hazard rates for PFS in the subgroup cohorts were calculated using the ratios (i.e., by multiplying the hazard rates for OS in the matched subgroup cohorts); finally, the hazard rates at each cycle were fitted with the Weibull hazard function to estimate the shape and scale parameters.

### Medical Costs and Utilities

The costs of each strategy (Table 1) were estimated from the perspective of the healthcare system in China. In this analysis, indirect costs were not included. Direct medical costs that were considered in the model included first- and second-line medical therapies, concomitant medication during therapy, the management of treatment-related severe adverse events (SAEs) (grade 3–4), routine follow-up, laboratory tests and BSC in terminally ill patients.

**Table 1:** Medical Resource Use and Costs Estimates ($, year 2009 values).

Parameter	Median Cost ($)	Range $)	Description and Reference
Operation	5,150§	3,680∼6,600	The overall cost acquired from local hospitals
Biopsy	1,180§	882∼1,470	The overall cost acquired from local hospitals
Radiotherapy	100 per fraction	90∼120	Local charge
Temozolomide			Shanghai development and reformation commission
100 mg	156	140∼171	
20 mg	38	34∼41	
Nimustine 25 mg	57	51∼62	Shanghai development and reformation commission
Second-line composite drug costs	125† per cycle	115∼135	Local charge
Supportive care	735* per cycle	480∼1,060	Local charge
Routine follow-up of patients	90‡ per unit	70∼120	Local charge
Serious adverse events			
Hematologic toxicity	321¶	289∼353	Local charge
Infection	588¶	529∼647	Local charge
Gastrointestinal toxicity	263¶	236∼289	Local charge

§ Components of costs were drugs and medical consumables (68%), surgery (10%), examination (9%), ward treatment and nursing (6%), anesthesia (4%) and accommodation and meals (3%).

† The cost included the chemotherapeutic agents (85%) and other adjuvant drugs (15%).

‡ The cost included the physician visit (1%), magnetic resonance imaging or computed tomographic scan (75%), other examinations and drugs (24%).

* The cost included caregiver (20%) and symptom-released drugs (80%).

¶ The cost included drugs and medical consumables (87%), ward treatment and nursing (7%), and accommodation and meals (6%).

The treatment costs were estimated according to the following schedules: 1) Radiotherapy: using a linear accelerator, a total dose of 60 Gy was delivered as the focal radiotherapy once daily at 2 Gy per fraction, 5 days/week; 2) TMZ: 75 mg/m^2^ per day during radiotherapy (concomitant chemotherapy), 4 weeks off, and then six cycles of 150–200 mg/m^2^ for 5 days every 28 days were administered (adjuvant chemotherapy); 3) Nitrosoureas: nimustine (ACNU) is a type of nitrosourea [14], [15] that is widely prescribed for GBM patients in China, so the cost of ACNU was used in the model as the cost of nitrosourea ACNU dosed at 100 mg/m^2^, which was assumed to be administered intravenously once every 6 weeks until the tumor progressed. The PCV regime in the second-line treatment was administered at 8-week intervals: lomustine 110 mg/m^2^ taken orally on day 1, procarbazine 60 mg/m^2^ taken orally on days 8 to 21, and vincristine 1.4 mg/m^2^ (maximal dose 2 mg) administered intravenously on days 8 and 29 [11]. To estimate the dosages of the chemotherapeutic agents, we assumed that a typical patient weighed 65 kg and had a height of 1.64 m, resulting in a body surface area of 1.72 m^2^ [16].

The EORTC-NCIC study showed that hematologic toxicity, gastrointestinal toxicity and infection were the main SAEs in the RT+TMZ combined strategy. Our model incorporated these treatment-related SAEs. SAE (grade≥3) management strategies were estimated based on patient records in local hospitals (Table 1). We assumed that the probabilities of the SAEs among the subgroup cohorts were similar to those of the overall cohorts.

The utility values for the various discrete health states were obtained from a previously published report [17] and are shown in Table 2. We assumed that the utilities of patients in China and the UK were equivalent. Because TMZ and nitrosoureas are both alkylating agents, we assumed that their utilities in RT+NT were equivalent to those in RT+TMZ. When the tumor progressed, the utility value in the state of the progressed disease would decrease by 0.02 per month.

**Table 2:** Base-Case Utilities.

State	Mean (range)	Reference
Progression-free	0.8872 (0.525–1.0)	[17]
Progression-free+RT	0.8239 (0.425–0.995)	[17]
Progression-free+RT+TMZ	0.7426 (0.175–0.98)	[17]
Progression-free+RT+NT	0.7426 (0.175–0.98)	[17]
Progression-free+TMZ	0.7331 (0.175–0.99)	[17]
Progression-free+NT	0.7331 (0.175–0.99)	[17]
Progressed	0.7314 (0.125–0.995)	[17]

### Economic Analyses

All parameters, including rates, costs and utilities, were entered into the model with a statistical distribution: lognormal distributions were assigned to all input costs; beta distributions were assigned to the utilities, probabilities and proportions; and bivariate normal distributions were assigned to all Weibull parameters. Using these distributions, a probabilistic sensitivity analysis (PSA) based on a Monte-Carlo simulation (1,000 simulations) was performed to evaluate the impact of uncertainty across all of the parameters simultaneously. Following WHO recommendations, we used 3× per capita GDP of China ($ 11,034)/QALY and 3× per capita GDP of Shanghai City ($ 38,376)/QALY as the threshold values [18]. Cost-effectiveness plane acceptability curves were plotted based on the outcomes projected from all 1,000 simulations, which estimated the willingness to pay (WTP) threshold for an incremental unit of effectiveness. The base-case analysis was run for 5 years, which was nearly a life-time horizon. Finally, to identify key model input parameters relating to the robustness of the results, one-way sensitivity analyses were conducted over the ranges shown in Tables S1, 1 and 2. The results are expressed as tornado charts.

## Results

### Validation of the Model

The base-case model compared the clinical outcomes to the results from clinical phase 3 trials. The median OS time from the trials, in addition to models for the overall cohort, and subgroups differentiated by age, MGMT methylation status and surgery status, are shown in Table 3. The model-estimated data were controlled at the 95% CI of the clinical trial data. The median PFS data measured in the overall cohort and in the subgroups stratified by the status of MGMT methylation were also set at the 95% CI of the trial data. This indicates that our method for estimating the missing PFS time data was a practical solution. Overall, these results validated the model.

**Table 3:** Trial Data and Model Estimated Values.

	Median OS times (months)			Median PFS times (months)		
Treatment arm	Trial (95% CI)	Model	Difference	Trial (95% CI)	Model	Difference
**Overall survival**						
RT	12.1 (11.2–13.0)	11.7	−0.4	5.0(4.2–5.5)	4.7	−0.3
RT+TMZ	14.6 (13.2–16.8)	14.9	0.3	6.9(5.8–8.2)	7.4	0.5
**MGMT methylated**						
RT	15.3 (13.0–20.9)	15.6	0.3	5.9 (5.3–7.7)	6.8	0.9
RT+TMZ	23.4 (18.6–32.8)	24.1	0.7	10.3 (6.5–14.0)	10.4	0.1
**MGMT unmethylated**						
RT	11.8 (10.0–14.4)	11.6	−0.2	4.4 (3.1–6.0)	4.0	−0.4
RT+TMZ	12.6 (11.6–14.4)	12.9	0.3	5.3 (5.0–7.6)	5.3	0.0
**Complete resection**						
RT	14.2 (12.1–16.1)	14.0	−0.2	-	5.6	NA
RT+TMZ	18.8 (16.4–22.9)	18.0	−0.8	-	8.7	NA
**Partial resection**						
RT	11.7 (9.7–13.1)	11.6	−0.1	-	4.6	NA
RT+TMZ	13.5 (11.9–16.4)	12.0	−1.5	-	7.1	NA
**Biopsy only**						
RT	7.8 (6.4–10.6)	7.8	0.0	-	3.6	NA
RT+TMZ	9.4 (7.5–13.6)	9.0	−0.4	-	4.4	NA
**Age <50 years**						
RT	13.6 (11.6–15.6)	12.7	−0.9	-	5.4	NA
RT+TMZ	17.4 (15.3–21.5)	15.1	−2.3	-	9.5	NA
**Age 50–60 years**						
RT	12.0 (10.0–14.2)	11.9	−0.1	-	5.2	NA
RT+TMZ	14.6 (13.6–17.9)	13.8	−0.8	-	6.4	NA
**Age >60 years**						
RT	11.8 (10.4–12.7)	11.8	0.0	-	4.3	NA
RT+TMZ	10.9 (8.9–14.9)	10.4	−0.5	-	6.1	NA

NA: not applicable.

### Base Case Analysis

The base case cost-effectiveness results (Table 4) were based on a 5-year time horizon. The model projected that TMZ resulted in a QALY of 1.09 in the overall cohort, which represents an increase of 0.29 QALYs over RT and an increase of 0.25 QALYs over NT. In the subgroup analysis, the results indicate a tendency for additional health benefits to be achieved for each treatment strategy in the cohort groups with more favorable prognostic factors. For example, the additional utilities gained by the RT, NT and TMZ strategies in subgroups with MGMT methylated, compared to subgroups with MGMT unmethylated, were 0.36, 0.48 and 0.6 QALYs, respectively.

**Table 4:** Base Case Results for the Alternative Strategies for Cost, QALY Gained and ICER.

	Cost ($)			Utility (QALY)			ICER		
Cohort	RT	NT+RT	TMZ+RT	RT	NT+RT	TMZ+RT	TMZ+RT VS. RT	TMZ+RT VS. RT+NT	NT+RT VS. RT
Overall cohort	7,234.0	8,655.9	32,562.4	0.80	0.84	1.09	87,940.6	94,968.3	39,185.1
Subgroups:									
MGMT methylated	7,753.3	9,500.0	37,598.2	1.12	1.27	1.47	7,015.3	141,144.1	11,237.3
MGMT unmethylated	7,206.6	8,540.0	31,319.8	0.76	0.79	0.87	19,188.7	299,673.0	46,454.0
Complete resection	7,677.3	9,678.5	34,644.4	0.94	1.02	1.27	6,898.8	100,108.0	26,207.2
Partial resection	7,267.0	8,703.3	28,193.7	0.80	0.84	0.96	11,180.8	168,668.1	35,537.2
Biopsy only	6,074.1	6,855.1	25,598.8	0.57	0.61	0.73	10,741.1	157,802.5	23,883.3
Age <50 years	7,672.3	9,900.1	29,351.4	0.87	0.97	1.16	6,125.4	103,010.5	20,995.6
Age 50∼60 years	7,374.4	8,866.0	32,182.4	0.82	0.86	1.02	10,609.3	155,206.9	33,419.7
Age >60 years	6,990.5	8,261.0	26,388.8	0.78	0.79	0.86	21,434.9	279,507.5	120,317.5

**Figure 2:**
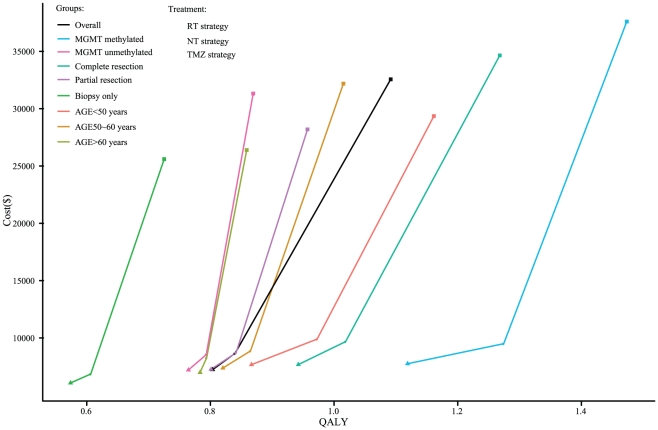
Analysis of the cost effectiveness of the first-line strategies for GBM in the overall cohort and the 8 subgroups. The x-axis represents the undiscounted 5-year quality-adjusted life-years (QALYs) for each strategy, and the y-axis represents the total undiscounted 5-year costs (in US dollars). The oblique line connects the RT strategy and the most cost-effective strategies; strategies above the straight lines were dominated or extended dominated.

Table 4 also shows the total direct costs in the overall cohort and in the eight risk-score subgroups. In the overall cohort, the total cost of the TMZ strategy was $ 32,562.4, followed by $ 8,655.9 for the NT strategy and $ 7,234.0 for the RT strategy. The TMZ strategy was the most expensive strategy. Subgroups with more favorable prognostic factors incurred higher direct medical costs. However, except in the partial resection and biopsy only subgroup, the TMZ strategy resulted in lower ICERs with more favorable prognostic factors compared to the RT and NT strategies (Table 4). This indicates that patients with more favorable prognostic factors would be more reasonable candidates for the TMZ strategy. Regardless of whether the analysis focused on the overall cohort or on one of the eight risk score subgroups, no NT strategy was dominated by the TMZ strategy because of the lower prices associated with the NT strategy (Figure 2).

### Uncertainty Analyses

**Figure 3:**
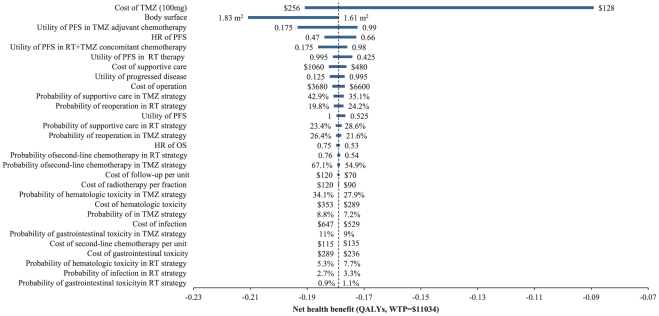
A tornado diagram of one-way uncertainty analyses in the overall cohort. The graph shows the effects of the variables on net health benefit (in QALYs, with WTP = $11,034) between the RT and TMZ strategies. The width of the bars represents the range of the results when the variables are changed, as shown in Tables 1, 2, 3. The vertical dotted line represents the base-case results. The vertical line represents the base-case value for the net health benefit with WTP = $11,034. PFS: progression-free survival; OS: overall survival; HR: hazard ratio.

The one-way sensitivity analyses reveal that some model parameters had a substantial impact on the net health benefit of the TMZ strategy compared to the RT strategy. The most influential parameter presented in the tornado graphs (Figure 3) was the cost of TMZ per 100 mg dose. Changing the cost for TMZ by an amount in a range from $ 128 to $ 256 had the effect of significantly changing the net health benefit. At the lower cost of TMZ, which resulted in a lower total cost for the TMZ strategy, the net health benefit increased to −1.07 QALYs (WTP = $11,034). A higher total cost for the TMZ strategy was observed at a higher cost for the TMZ, with the net health benefit decreasing to −2.29 QALYs. In this analysis, the patients were assumed to have a body surface area of 1.72 m^2^. This estimate was determined using a range from 1.61 to 1.83 m^2^. The body surface area was thus determined to be the second most substantial effect factor, leading to a change in the net health benefit from −2.15 to −2.53 QALYs. The other important effect factors of the model were the utility of the PFS in TMZ adjuvant chemotherapy and the HR for the TMZ strategy versus the RT strategy. Other factors, such as the costs of managing SAEs and incidences of SAEs, had little impact (not shown in the tornado diagram).

**Figure 4:**
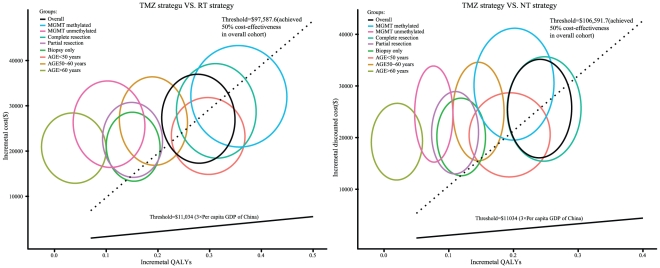
The probabilistic results of the incremental cost-utility differences for GBM in the overall cohort and the 8 subgroups. The TMZ strategy was compared to: (A) the RT strategy and (B) the NT strategy for a cohort of 1,000 GBM patients. The y-axis represents the incremental costs. The x-axis represents the incremental QALYs gained. Each ellipse represents the 95% confidence interval ellipse of the probabilistic results. The proportion of the ellipses found below the ICER threshold (the oblique lines) reflects the simulations in which the cost per additional QALY gained with the TMZ strategy was below the ICER threshold.

The PSA comprising 1,000 simulations measured the probabilities of meeting the ICER thresholds of $11,034 and $38,376 per additional QALY for the TMZ strategy compared to RT and NT strategies for GBM patients. The results are presented in Figure 4. When the ICER threshold was $11,034, the probabilities of achieving cost-effectiveness with the TMZ strategy relative to that with the RT and NT strategies in the overall cohort and the 8 risk score subgroups were all zero. When the threshold increased to the point where there was a 50% probability of achieving cost effectiveness in the overall cohort, the subgroups with MGMT methylated, complete resection and age<50 years achieved greater than 50% cost-effectiveness with the TMZ strategy compared to the RT strategy, and the subgroups with complete resection and age<50 years gained greater than 50% cost-effectiveness with the TMZ strategy compared to the NT strategy.

**Figure 5:**
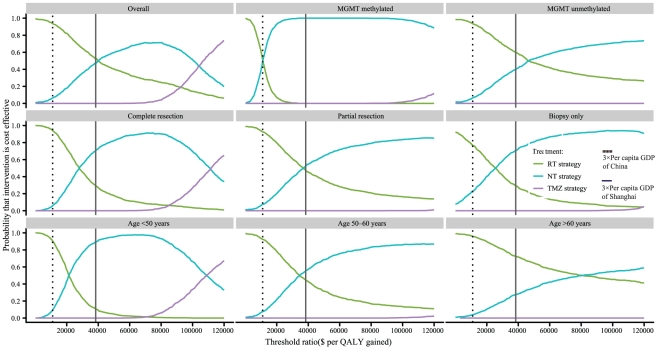
The cost-effectiveness acceptability curves for the three first-line strategies for GBM in the overall cohort and the 8 subgroups. The vertical axes represent the probabilities of cost effectiveness. The horizontal axes represent the willingness-to-pay thresholds to gain 1 additional quality-adjusted life-year (QALY). The bold vertical dashed and solid lines represent the thresholds for China and Shanghai City, respectively.

The cost-effectiveness acceptability curves (CEACs) show the preferred first-line strategies for GBM in the overall cohort and the 8 subgroups when a range of cost-per-QALY thresholds is taken into account. The CEAC plot shows that when the threshold was $11,034, the likelihood of achieving cost-effectiveness with the RT strategy might be higher than the likelihood of achieving cost-effectiveness with the TMZ and NT strategies in the overall cohort and in 7 subgroups. In the subgroup with MGMT methylated patients, the NT strategy achieved a similar probability of cost-effectiveness as the RT strategy. When the threshold was $38,376, the NT strategy achieved the maximum likelihood of cost-effectiveness in the subgroups with MGMT methylated, complete resection, partial resection, biopsy only, age<50 years and age 50∼60 years (Figure 5).

## Discussion

Since TMZ was introduced as a first-line treatment in newly diagnosed GBM, survival rates and quality of life have both improved. However, the widespread use of TMZ has resulted in a dramatic increase in healthcare costs. An economic evaluation of the recommended TMZ strategy as a first-line therapy in a health resource–limited setting can help policy-makers, physicians and patients to make the proper decisions. Using decision-analytic modeling techniques, we estimated the cost-effectiveness of three first-line GBM strategies in an overall cohort and in 8 subgroups with different prognostic factors from the perspective of the Chinese healthcare system over a 5-year period.

Our results suggest that the TMZ strategy as a first-line treatment for GBM may possess significant advantages in relation to health benefits. However, the gap between the costs of TMZ and payment capacity in a health resource-limited setting may be too great to allow the TMZ strategy to be recognized as the most appropriate approach. In the overall cohort, the TMZ strategy (when compared to the RT and NT strategies) revealed benefits that were achieved at an incremental cost per QALY of $ 87,940.6 and $ 94,968.3, respectively. These costs are far greater than the societal willingness-to-pay thresholds for each additional quality-adjusted life-years gained ($11,034 and $38,376 for all of China and Shanghai City, respectively). These higher ratios are largely attributable to the high cost and the relatively limited survival benefits associated with TMZ. The ICERs identified in other health economic analyses from relatively health resource-rich regions ranged from €37,000 per life-year gained to €42,840 per QALY gained for the TMZ strategy compared with the RT strategy [19], [20], [21]. These results indicate that the addition of TMZ to radiotherapy in GBM patients is relatively inefficient in economic terms. It was expected that a major cost reduction might be achieved if the use of TMZ was restricted to those patients who had a greater likelihood of benefiting from it [22]. Our subgroup cohort analyses with three major prognostic factors indicated that the ICERs of TMZ, when compared to the RT and NT strategies in the eight subgroups, gradually decrease as the prognostic factors become more favorable. However, no ICERs could achieve the feasible affordability threshold for society in China or in Shanghai City. Compared to the TMZ and NT strategies, the RT strategy provided a higher probability of cost-effectiveness under the Chinese threshold in the overall cohort and in almost all of the subgroups. The NT strategy seemed to be a cost-effective option under the threshold of Shanghai City for 6 subgroups when compared to the TMZ and RT strategies. Different regions should consider different therapies for GBM based on their capacities for affording the costs. Chinese physicians and patients often face dilemmas regarding the use of expensive therapies. The TMZ strategy also faces this dilemma because it is an expensive treatment option. To a certain extent, it might still be appropriate to recommend the TMZ strategy for some subgroups with a lower ICER for the TMZ strategy compared to the RT and NT strategies. Overall, our results indicate that the strategy for treating GBM should carefully consider the results of economic analysis to optimize the allocation of health resources, especially in resource-limited settings.

The one-way sensitivity analysis shows that the results of the model were driven by certain key parameters (particularly the cost of TMZ). As Figure 4 shows, when the cost of TMZ was increased or decreased within the range of the upper and lower prices of TMZ, the net health benefit increased or decreased dramatically. The absolute value of the difference reached 1.22 QALYs. This finding indicates that the pharmaceutical industry should adopt a more prudent and conservative approach in its pricing [22]. At present, a generic version of TMZ is being supplied for Chinese patients at a price that is approximately half the price of the brand-name TMZ drug. If the clinical efficacy of the generic drug is equal to that of the brand-name drug, the cost-effectiveness of the TMZ strategy will improve significantly. Because the dosages of TMZ administered to the patients were calculated using the body surface area, the body surface area was found to be the second most influential factor. A higher body surface area resulted in a higher dose of TMZ, which in turn increased the cost of the TMZ strategy and ICERs compared to the RT strategy. Other important influential factors include the utility of PFS in TMZ adjuvant chemotherapy, the HR of PFS in the TMZ strategy compared to the RT strategy and the utility of PFS in RT+TMZ concomitant chemotherapy. Other factors, such as the probabilities of SEAs and the costs of managing SEAs, did not significantly affect the final results.

Several limitations of the current analysis must be considered. First, the estimation of the Weibull parameters of PFS for the subgroups of age and surgery type was an inevitable limitation. The Weibull parameters of PFS determined the survival rates of the disease-free patients. However, the results of the comparison of the median PFS times between the model and the trial in the MGMT subgroups indicates that the estimation method used in this study minimized this bias. Second, because few head-to-head trials have been conducted for the three first-line strategies for GBM, an indirect comparison was used in this study. Similar patient characteristics for the three strategies were assumed in our indirect comparison, and the results of the indirect comparison were imputed into the analytical model. When there is no direct comparison trial, indirect comparisons using well-recognized methods have been accepted by many researchers around the world. When direct comparison data are available, this analysis could be updated. Third, we did not fully explore other therapeutic strategies for first- and second-line treatments of GBM, especially targeted therapies such as bevacizumab [23], [24], which could improve the survival rates, although at a high cost. If a strategy is able to prolong the PFS time, the cost of the second-line treatment will be reduced. Because the targeted therapies are considered in the second-line treatment, the ICER of the TMZ strategies could be improved. Fourth, the choice of the Chinese healthcare system as our baseline perspective was narrow; as a result, only the direct medical costs were estimated in the analysis. An overall societal perspective that encompasses the indirect costs of the disease, such as the burden on the families and caregivers, may expand the costs associated with GBM. As such, oral medications (e.g., TMZ) and prolonged PFS (e.g., TMZ) may produce more favorable results. However, there is no well-established method for incorporating such indirect costs into the analysis when measuring the cost effectiveness of first-line therapies for GBM. Finally, utility values were derived from literature published abroad because of the absence of Chinese data on this issue. However, we believe that this analysis can provide helpful information for Chinese health policy decision-makers because the results of this analysis reflect the general practice for treating newly diagnosed GBM in China.

In conclusion, in the Chinese healthcare setting (which is representative of a health resource-limited region), the addition of TMZ to radiotherapy in patients with GBM would not be a cost-effective approach compared to radiotherapy alone or radiotherapy plus nitrosourea agents. However, better economic outcomes are likely to occur when subgroups with more favorable prognostic factors receive TMZ. Decreasing the price of TMZ might be one potential way to counter the restrictive Chinese reimbursement policies.

## Supporting Information

Table S1 **Baseline Clinical Data.**
(DOCX)
