# Supplementary material for: Subgroup Economic Analysis for Glioblastoma in a Health Resource-Limited Setting
Source: PLoS One. 2012 Apr 12;7(4):e34588. doi: 10.1371/journal.pone.0034588 (PMC3325281; doi:10.1371/journal.pone.0034588)
Supplement: Table S1 — Baseline Clinical Data. (DOCX) [file pone.0034588.s001.docx]

| Table S1. Baseline Clinical Data |  |  |  |  |
| --- | --- | --- | --- | --- |
| **Variables** | **Base-case parameters of Weibull Curves** | | | **Reference** |
|  | **Scale values (SD)** | **Shape values (SD)** | **Adjusted R^2^** |  |
|  |  |  |  |  |
| Overall survival |  |  |  |  |
| Overall cohort |  |  |  |  |
| RT | 0.01047(0.00096) | 1.703(0.034) | 0.9947 | [[7](#_ENREF_7)] |
| RT+NT | 0.00890(0.00082) | 1.703(0.034) | - | calculated |
| RT+TMZ | 0.03978(0.00413) | 1.057(0.032) | 0.973 | [[7](#_ENREF_7)] |
| MGMT methylated |  |  |  |  |
| RT | 0.008839(0.00119) | 1.589(0.044) | 0.9867 | [[7](#_ENREF_7)] |
| RT+NT | 0.006983(0.00094) | 1.589(0.044) | - | calculated |
| RT+TMZ | 0.01132(0.00073) | 1.293(0.018) | 0.9942 | [[7](#_ENREF_7)] |
| MGMT unmethylated |  |  |  |  |
| RT | 0.0007228(0.00008) | 2.805(0.041) | 0.9984 | [[7](#_ENREF_7)] |
| RT+NT | 0.00057 (0.00008) | 2.805(0.041) | - | calculated |
| RT+TMZ | 0.000434(0.00008) | 2.805(0.041) | 0.9122 | [[7](#_ENREF_7)] |
| Complete resection |  |  |  |  |
| RT | 0.007021(0.01241) | 1.741(0.605) | 0.9939 | [[7](#_ENREF_7)] |
| RT+NT | 0.00547638(0.00968) | 1.741(0.605) | - | calculated |
| RT+TMZ | 0.03391(0.02771) | 1.044(0.2385) | 0.9844 | [[7](#_ENREF_7)] |
| Partial resection |  |  |  |  |
| RT | 0.01255(0.01604) | 1.635(0.47) | 0.9888 | [[7](#_ENREF_7)] |
| RT+NT | 0.0106675(0.01363) | 1.635(0.47) | - | calculated |
| RT+TMZ | 0.08036(0.12263) | 0.8659(0.4652) | 0.9162 | [[7](#_ENREF_7)] |
| Biopsy only |  |  |  |  |
| RT | 0.05361(0.05028) | 1.248(0.4025) | 0.9889 | [[7](#_ENREF_7)] |
| RT+NT | 0.04782012(0.04485) | 1.248(0.4025) | - | calculated |
| RT+TMZ | 0.07139(0.1023) | 1.033(0.5089) | 0.9485 | [[7](#_ENREF_7)] |
| Age <50 years |  |  |  |  |
| RT | 0.01683(0.03912) | 1.464(0.7984) | 0.9491 | [[7](#_ENREF_7)] |
| RT+NT | 0.0124542(0.02895) | 1.464(0.7984) | - | calculated |
| RT+TMZ | 0.09401(0.09372) | 0.7356(0.2871) | 0.949 | [[7](#_ENREF_7)] |
| Age 50–60 years |  |  |  |  |
| RT | 0.01371(0.0155) | 1.583(0.407) | 0.9906 | [[7](#_ENREF_7)] |
| RT+NT | 0.0116535(0.01318) | 1.583(0.407) | - | calculated |
| RT+TMZ | 0.0354(0.04825) | 1.132(0.4274) | 0.9659 | [[7](#_ENREF_7)] |
| Age >60 years |  |  |  | calculated |
| RT | 0.005278(0.00738) | 1.977(0.534) | 0.9928 | [[7](#_ENREF_7)] |
| RT+NT | 0.00459186(0.00642) | 1.977(0.534) | - | calculated |
| RT+TMZ | 0.09119(0.05877) | 0.8647(0.2038) | 0.9845 | [[7](#_ENREF_7)] |
| **Progression-free survival** |  |  |  |  |
| Overall cohort |  |  |  |  |
| RT | 0.08062(0.00343) | 1.391(0.021) | 0.9938 | [[12](#_ENREF_12)] |
| RT+NT | 0.06691(0.00285) | 1.391(0.021) | - | calculated |
| RT+TMZ | 0.08597(0.00355) | 1.043(0.016) | 0.9893 | [[12](#_ENREF_12)] |
| MGMT methylated |  |  |  | calculated |
| RT | 0.1493(0.0164) | 0.9005(0.044) | 0.8803 | [[13](#_ENREF_13)] |
| RT+NT | 0.1179(0.0130) | 0.9005(0.044) | - | calculated |
| RT+TMZ | 0.05141(0.00135) | 1.124(0.01) | 0.9966 | [[13](#_ENREF_13)] |
| MGMT unmethylated |  |  |  |  |
| RT | 0.05151(0.00314) | 1.733(0.034) | 0.9928 | [[13](#_ENREF_13)] |
| RT+NT | 0.04121(0.00314) | 1.733(0.034) | - | calculated |
| RT+TMZ | 0.03234(0.00228) | 1.687(0.033) | 0.9915 | [[13](#_ENREF_13)] |
| Complete resection |  |  |  |  |
| RT | 0.0538(0.01241) | 1.429(0.605) | 1 | calculated |
| RT+NT | 0.0447(0.0103) | 1.429(0.606) | - | calculated |
| RT+TMZ | 0.0733(0.02771) | 1.03(0.2385) | 1 | calculated |
| Partial resection |  |  |  |  |
| RT | 0.09755(0.01604) | 1.323(0.47) | 1 | calculated |
| RT+NT | 0.08097(0.01331) | 1.323(0.48) | - | calculated |
| RT+TMZ | 0.1742(0.12263) | 0.8519(0.4652) | 1 | calculated |
| Biopsy only |  |  |  |  |
| RT | 0.4496(0.05028) | 0.936(0.4025) | 1 | calculated |
| RT+NT | 0.3732(0.04173) | 0.936(0.4026) | - | calculated |
| RT+TMZ | 0.1543(0.1023) | 1.019(0.5089) | 1 | calculated |
| Age <50 years |  |  |  |  |
| RT | 0.1345(0.03912) | 1.152(0.7984) | 1 | calculated |
| RT+NT | 0.1116(0.03247) | 1.152(0.7985) | - | calculated |
| RT+TMZ | 0.2044(0.09372) | 0.7216(0.2871) | 1 | calculated |
| Age 50–60 years |  |  |  |  |
| RT | 0.1074(0.0155) | 1.271(0.407) | 1 | calculated |
| RT+NT | 0.0891(0.0129) | 1.271(0.408) | - | calculated |
| RT+TMZ | 0.07644(0.04825) | 1.118(0.4274) | 1 | calculated |
| Age >60 years |  |  |  |  |
| RT | 0.03942(0.00738) | 1.665(0.534) | 1 | calculated |
| RT+NT | 0.03272(0.00613) | 1.665(0.535) | - | calculated |
| RT+TMZ | 0.1977(0.05877) | 0.8507(0.2038) | 1 | calculated |
|  |  |  |  |  |
|  | **Base-case proportions*** **(range) of treatment arms** | | |  |
| **Second-line treatment** | **RT** | **RT+NT** | **RT+TMZ** |  |
| Reoperation | 0.22(0.165~0.275) | 0.24(0.18~0.3) | 0.24(0.18~0.3) | [[7](#_ENREF_7)] |
| Chemotherapy-based composite treatment | 0.74(0.555~0.925) | 0.61(0.4575~0.7625) | 0.61(0.4575~0.7625) | [[7](#_ENREF_7)] |
| Supportive care only | 0.26(0.195~0.325) | 0.39(0.2925~0.4875) | 0.39(0.2925~0.4875) | [[7](#_ENREF_7)] |
| **Main severe toxicity (3-4 grade)** |  |  |  |  |
| Hematologic toxicity | 0.07(0.0525~0.0875) | 0.31(0.25~0.64) | 0.14(0.105~0.175) | [[12](#_ENREF_12)] |
| Gastrointestinal toxicity | 0.01(0.0075~0.0125) | 0.1(0.075~0.125) | 0.02(0.015~0.025) | [[12](#_ENREF_12)] |
| Infection | 0.03(0.0225~0.0375) | 0.08(0.06~0.1) | 0.07(0.0525~0.0875) | [[12](#_ENREF_12)] |

* All values are not percentages unless otherwise indicated.
